# Comment on “Assessment of Liver Stiffness in Pediatric Fontan Patients Using Transient Elastography”

**DOI:** 10.1155/2016/9343960

**Published:** 2016-11-29

**Authors:** Xiulan Xue, Shaohang Cai

**Affiliations:** ^1^First Affiliated Hospital of Xiamen University, Xiamen, Fujian Province, China; ^2^Department of Pathology, Sun Yat-sen University Cancer Center, Guangdong 510515, China

We read with great interest the study conducted by Chen et al. [1]. The authors demonstrated that transient elastography (TE) is a nonpainful, convenient, and safe way to assess the liver injury after Fontan surgery. The data indicated that TE values were higher in Fontan patients versus controls (18.6 versus 4.7 Kpa, *P* < 0.001), although there was no association between TE values and patients age, time since Fontan surgery, or median Fontan circuit pressure. The study design was elegant and the finding was interesting. However, we may wonder whether the liver stiffness among Fontan patients may be biased by alanine aminotransferase (ALT).

Numerous studies have examined the performance of TE in assessment of hepatic fibrosis. Among patients with normal ALT and bilirubin level, when TE value is more than 7.4 to 7.8 Kpa, the sensitivity and specificity to diagnosis of liver significant fibrosis (>*F*2) were 76–83% and 82–84%, and when TE value is more than 11.9 to 14.8 Kpa, the sensitivity and specificity to diagnosis of liver cirrhosis were 87–94% and 91–93% [2–4] (Table 1). In Chen's work, some Fontan patients' liver stiffness was higher than 30 Kpa, which seriously deviated from liver cirrhosis of general population. This intriguing result may be cause by the ALT levels. Studies have reported that performance of TE for fibrosis assessment was influenced seriously by ALT level and bilirubin level [5–7]. Liver stiffness value was well correlated with serum ALT level and higher liver stiffness was associated with higher serum ALT level. In Chen's work, the ALT levels among Fontan patients in the study were average 31.5 U/L from 12.0 to 54 U/L, significantly higher than control group with average 17.0 U/L from 12.0 to 32.0 U/L. That may bias the TE value. Hence, the reason why liver stiffness among Fontan patients was higher than control group may be not only because the level of liver fibrosis in Fontan patients is more severe than control group, but also because the ALT level is significantly higher in Fontan patients compared with control group. And that may explain the reason why TE value was not associated with time since Fontan surgery is with only *P* value of 0.06. Study has suggested that grouping patients according to ALT level is necessary for TE detection [8]. Although data from the study indicated that TE was efficacious for detecting liver fibrosis after Fontan surgery, stratifying patients according to ALT levels and comparing with control group with a similar ALT level may be much more convincing and interesting.

However, data from the study have proven that TE was convenient and efficacious detection to diagnosis of liver fibrosis among Fontan patients. That is a valuable suggestion for real time clinical practice. Until now, studies focusing on monitoring the progress of liver fibrosis in patients after Fontan surgery by TE detection are limited and an optimal cut-off value of liver stiffness is missing. A prospective clinical trial is needed to find an optimal cut-off value to diagnosis of the progress of liver fibrosis after Fontan surgery. So clinical physician can take a timely medical intervention for those Fontan patients with serious liver fibrosis to prevent disease progress to end-stage liver disease, such as liver cirrhosis and hepatocellular carcinoma.

## Figures and Tables

**Table 1:** Performance of transient elastography for detection advanced fibrosis and liver cirrhosis.

Author	Patients	Advanced fibrosis (>*F*2)			Liver cirrhosis		
		Cut-off	Se%	Sp%	Cut-off	Se%	Sp%
Lupsor et al. 2008	324	7.4	76	84	11.9	87	91
Arena et al. 2008	150	7.8	83	82	14.8	94	92
Platon et al. 2013	1202	7.4	80	84	13.2	94	93

Se, sensitivity; SP, specificity.
